# *Typhlodromuspyri* and *Euseius finlandicus* (Acari: Phytoseiidae) as potential biocontrol agents against spider mites (Acari: Tetranychidae) inhabiting willows: laboratory studies on predator development and reproduction on four diets

**DOI:** 10.1007/s10493-015-9973-5

**Published:** 2015-11-03

**Authors:** Ewa K. Puchalska, Marcin Kozak

**Affiliations:** Department of Applied Entomology, Faculty of Horticulture, Biotechnology and Landscape Architecture, Warsaw University of Life Sciences-SGGW, Warsaw, Poland; Department of Botany, Faculty of Agriculture and Biology, Warsaw University of Life Sciences-SGGW, Warsaw, Poland

**Keywords:** Life table, Biological control, Alternative food, Development, Predator, Pollen

## Abstract

*Typhlodromuspyri* Scheuten and *Euseius finlandicus* (Oudemans) are important predators of phytophagous mites. The present laboratory study aimed to determine whether both species can develop and reach maturity feeding on spider mites occurring on willows, i.e., *Schizotetranychus schizopus* (Zacher), *Schizotetranychus garmani* Pritchard & Baker, and *Tetranychus urticae* Koch, and on *Brassica napus* L. pollen. The predators’ development, reproduction and demographic parameters were significantly affected by diet. The data suggest that rape pollen can be useful in mass rearing of *E. finlandicus* but is completely unsuitable as alternative food for *T*. *pyri*. Short development time and high values of population parameters achieved by *T. pyri* feeding on larvae and protonymphs of *S. schizopus* and by *E. finlandicus* feeding on juvenile stages of *S. garmani* indicate great suitability of these preys as food for the phytoseiids, and make both predatory species promising biocontrol agents in spider mite control on willows.

## Introduction

Plants of the genus *Salix* are grown for energy purposes (Gigler et al. 1999; Johansson and Lundqvist 1999) and for the chemical industry (cellulose, bio-alcohols; Cook and Beyea 2000) and furniture industry (Sean and Labrecque 2006; Warmbier et al. 2010). Willows also have a high ecological value; for example, they are used to form protection zones along the routes (motorways) against noise or emissions of pollutants, and for reclamation of areas degraded and destroyed by industrial activities (Waliszewska et al. 2011). Several species and cultivars of willow are grown in ornamental nurseries and offered as a decorative element of landscape architecture.

Among many phytophagous arthropods occurring on cultivated willows, spider mites (Tetranychidae), i.e., *Schizotetranychus schizopus* (Zacher), *Schizotetranychus garmani* Pritchard & Baker and *Tetranychus urticae* Koch, are considered key pests (Soika and Łabanowski 2003; Tomczyk 2004; Puchalska et al. 2014). Difficulties of these pests’ management result from the rapid development of resistance to many acaricides used for their control. High fecundity, inbreeding, very short life-cycle, and high mutation rate of tetranychids facilitate resistance development and often induce a high degree of cross-resistance to pesticides (Cranham and Helle 1985; Van Leeuwen et al. 2009). Therefore, it seems reasonable to seek alternatives to chemical control of spider mites, including those occurring on willows. One of the possibilities is using biological agents.

The most important natural enemies of tetranychids are phytoseiid mites (McMurtry and Croft 1997). Nevertheless, biological control of spider mites with the use of phytoseiids is mainly based on introducing exotic species into greenhouses (Naher and Haque 2007; Oliveira et al. 2009). So far, only few attempts have been made to introduce predatory mites on ornamental plants in outdoor cultivation (Pratt et al. 2002; Shrewsbury and Hardin 2003; Kaźmierczak 2004). Releasing exotic natural enemies, especially in open-field cropping systems, may fail, e.g., due to lack of environmental adaptation or insufficient synchronization of life cycles of the agent and target pest (Gurr and Wratten 2000). In such a case releasing indigenous species of predators gives better results in pest control and also reduces potential risk of negative effects on non-target species and ecosystem in which the species function (Lockwood et al. 2001; van Lenteren et al. 2003; Wajnberg et al. 2007).

Observations of the Phytoseiidae complex occurring on willows, both in forests and ornamental plant nurseries, were conducted in Central Europe (Poland) by Puchalska et al. (2014). The authors stated that the dominant phytoseid species in natural conditions was *Euseius finlandicus* (Oudemans), whereas in nurseries willows were most frequently inhabited by *Typhlodromuspyri* Scheuten. Both the species are considered to be important predators of phytophagous mites in European orchards and vineyards (Schruft 1985; Maixner 1990; Abdallah et al. 2001). Their positive association with spider mites occurring on cultivated willows (Puchalska et al. 2014) indicates their potential role in pest management in ornamental nurseries. Data concerning developmental and reproductive parameters of both mentioned natural enemies feeding on their potential preys may provide important information into pest management programs. These parameters, especially the intrinsic rate of increase (r_m,_), are increasingly used as a means of selecting promising biocontrol candidates (Roy et al. 2003). Laboratory studies on development and reproduction of *T. pyri* and *E. finlandicus* feeding on different preys, i.e., tenuipalpids, eriophyoids, tydeids or even scale insects or whiteflies (Amano and Chant 1986; Duso and Camporese 1991; Schausberger 1998; Nomikou et al. 2001; Vargas and Cardemil 2005; Lorenzon et al. 2012) were previously conducted revealing information on predator–prey relationships. Several investigations were performed to determine life parameters of these predators fed on tetranychids such as *Panonychus ulmi* (Koch), *Eotetranychus tiliarium* (Hermann), *Eotetranychus carpini* (Oudemans) and *Te. urticae* (Zaher and Shehata 1971; Kropczyńska et al. 1988; Duso and Camporese 1991; Lorenzon et al. 2012). However, life histories of *T. pyri* and *E. finlandicus* reared on spider mites obtained from willows have yet to been studied. Those findings may improve knowledge useful in biological control of these key pests of ornamental nurseries. *Typhlodromus pyri* is classified as type III lifestyle—generalist predator and *E. finlandicus* as type IV—pollen feeding generalist predator (McMurtry et al. 2013). It is known that both these phytoseiids are able to successfully develop on pollen of different plant species (Duso and Camporese 1991; Schausberger 1992; Broufas and Koveos 2001; Lorenzon et al. 2012). Therefore, we decided to include in the experiments rape pollen as potential non-prey food. European Union is one of the major producers of oilseed rape (*Brassica napus* L.) (Carré and Pouzet 2014), with steadily increasing production area, thereby making the plant pollen easily available. Although the rape pollen is entomophilous, it can be airborne up to 1–1.5 km (Timmons et al. 1995, Devaux et al. 2005). Moreover, the pollen has a great nutritional quality for some arthropods especially due to high content of proteins and essential amino acids (Cook et al. 2003, Keller et al. 2005). A positive effect of rape pollen on *E. finlandicus* and/or *T*. *pyri* development could indicate its ability for rearing of the phytoseiids.

The aims of the present study were (1) to determine whether *T*. *pyri* and *E. finlandicus* can develop and reach maturity fed on spider mites occurring on willows, i.e., *S. schizopus*, *S. garmani* and *Te. urticae*, and (2) to assess effects of different diets (the three spider mites species and rape pollen) on the predators’ development, reproduction and demographic parameters.

## Materials and methods

### Plant material

Three-year old (counting from grafting) and 1.2 m high trees of goat willow (*Salix caprea*) cv. Kilmarnock were grown in plastic pots (30 cm diameter, 40 cm deep) filled with garden soil. Plants were watered on alternate days. Willows were divided to four groups kept in separate walk-in plant growth rooms (25 ± 5 °C, 65 ± 10 % RH and 16L:8D photoperiod). One of the rooms contained only uninfested plants. On trees kept in the remaining three rooms, three different spider mite species were cultured separately. Four willows were placed in each plant growth room, close to each other to allow spider mites to move freely between plants.

### Phytophagous mites rearing

*Schizotetranychus schizopus* was obtained from *S. caprea* cv. Kilmarnock leaves, and *Te. urticae* from *S. caprea* cv. Pendula leaves from ornamental plant nurseries situated in central Poland. A stock colony of *S. garmani* was established with individuals collected from *S. caprea,* grown in Powsin forest, in central Poland. About 60 females of each spider mite species were placed separately in modified Munger cells (as described below) for oviposition. After laying eggs, females were slide-mounted in Heinze PVA medium for phase contrast microscopic examination (Walter and Krantz 2009). The progeny of each spider mite species was then reared on detached willow leaves (Kilmarnock) placed upside-down directly on a wet sponge in open plastic trays containing water. Mites were reared for two generations in environmental chambers (Sanyo MLR-350) at 25 ± 0.5 °C, 70 ± 10 % RH and 16L:8D photoperiod. Then leaves with the respective spider mite species were transferred on willow trees placed in plant growth rooms (each species to a separate room). Every 3 weeks one uninfested tree was added to each room to maintain the culture.

### Stock colony of *Euseius finlandicus* and *Typhlodromus* (*T.*) *pyri*

*Euseius finlandicus* was collected from *S. caprea* trees growing in Łomianki forest in central Poland. The stock colony of *T. pyri* was initiated with specimens obtained from the Research Institute of Pomology and Floriculture in Skierniewice, Poland, where the mites were reared under laboratory conditions (24 ± 2 °C, 70 ± 10 % RH and 16L:8D photoperiod) on *Phaseolus vulgaris* L. plants infested by *Te. urticae.* Detached common bean plants bearing mites were transferred in refrigeration container to the Warsaw University of Life Sciences-SGGW (Warsaw, Poland).

Separate stock colonies of *E. finlandicus* and *T. pyri* were then maintained in the laboratory of the Department of Applied Entomology (SGGW) in the environmental test chambers (25 ± 5 °C, 70 ± 10 % RH and 16L:8D photoperiod). Using a fine paint brush, the predators were placed on detached willow leaves (Kilmarnock) lying on four layers of filter paper, which rested on a wet sponge 13 × 13 cm in an open plastic box (14 × 17 × 6.5 cm). To keep the sponge wet and to prevent the mites from escaping, water was added to the boxes. Additionally strips of wet tissue paper were placed on leaves to provide water. Rape pollen (*Brassica napus* L.) or all stages of *Te. urticae* or *S. schizopus* or *S. garmani,* obtained from the cultures described above, were provided to the predators as food. The pollen of *B. napus* plants grown in an ecological apiary (Lower Silesia Province, Poland) was collected in June. Bottom pollen traps (Dadant, USA) were used for receiving pollen pellets from legs of honey bees. Then the pollen was dried and stored in a freezer (−18 °C). For the experiments it was thawed and kept in a refrigerator at 4 °C for maximum 1 week. Mites were reared at these conditions for at least four generations before conducting the experiment.

### Experimental rearing units

All experiments were performed in environmental chambers (Sanyo MLR-350) at 25 ± 0.5 °C, 70 ± 10 % RH and 16L:8D photoperiod. We applied the method of individual rearing of phytoseiid mites on non-infested and fully expanded detached leaves of *S. caprea* ‘Kilmarnock’. The experimental unit was a modified Munger cell (Overmeer 1985). The cell consisted of a stack of four 100 × 50 mm layers, in the following order: 2 mm thick bottom Plexiglas plate covered with tissue paper, a detached willow leaf placed upside-down on the tissue paper, 7 mm thick plate with a 30 mm hole in the center sealed with plasticine and 2 mm thick top plate with a 10 mm ventilation hole, covered with muslin mesh. The plasticine was used to prevent mites from escaping from the arena. The stack was held together with rubber bands. To maintain humidity in the cell, the tissue paper was moistened daily with distilled water. In each unit, the predators were provided with surplus amounts of the following food items: (1) larvae and protonymphs of *Te. urtice*, (2) larvae and protonymphs of *S. schizopus*, (3) larvae and protonymphs of *S. garmani* or (4) rape pollen. Preys were refreshed twice a day with larval stages of tetranychids. Pollen was replenished every day with a fine paint brush, about 0.5 mg per day. A piece of a transparent plastic sheet folded in the shape of a tent was placed over each arena as a shelter and oviposition site for phytoseiids. Once every 9 days *E. finlandicus* and *T. pyri* individuals were transferred to a new experimental units with the appropriate prey, as previously described, to avoid an effect of leaf aging.

### Life history experiment

Twelve hours before the experiment gravid females of both phytoseiid species were transferred from a stock colonies to separate rearing units to obtain eggs of similar age. After that time eggs were placed individually in the Munger cells described above. Thirty replicates per combination of Phytoseiidae species and diet were used. To determine the duration and survivorship of each life stage of *E. finlandicus* and *T. pyri*, observations were made every 24 h until all individuals had reached adulthood. The presence of an exuvium was used to establish successful molting to the next developmental stage.

After completing the immature development, females were transferred to new experimental units with the appropriate food and paired with a male. When the male died or escaped, a new one was added from the respective stock colony. Fifteen replicates for each predator/food combination were used. The experimental units were examined every 24 h to determine the duration periods of pre-oviposition, oviposition, post-oviposition as well as longevity and fecundity of females. The eggs laid the same day were placed in a single unit and reared to adulthood to determine sex-ratio of the progeny.

### Data analysis

Influence of phytoseiid species and food item on immature developmental time, female longevity, duration of pre-oviposition, oviposition and post-oviposition was studied by two-way analysis of variance, which also included species-by-food interaction. The fitted models were checked graphically (Quinn and Keough 2002); in all the situations the fit was sufficient. Since in all the analyses the interaction between the predator species and food source was statistically significant, multiple comparisons were conducted for combinations of these two factors, without adjustment for multiple testing (Webster 2007, Kozak 2009).

Comparison of female fecundity on different diets was done by means of generalized linear models, with Poisson distribution of the residuals. Since the variability in the data when compared to the mean of the Poisson distribution was too small, quasi generalized linear modeling was employed. For a two-way model (with predator species and food item as factors) a significant interaction was observed, so again pair-wise comparisons for combinations of the two factors was employed, without adjustment for multiple testing (Webster 2007; Kozak 2009).

Life tables were constructed from the observed age-specific survival rate (*l*_*x*_) and age-specific fecundity rate (*m*_*x*_) [net reproductive rate (*R*_0_), mean generation time (*T*), intrinsic rate of population increase (*r*_*m*_) and finite rate of population increase (*λ*)] (Birch 1948). Standard errors of the population parameters, estimated for the studied phytoseiids reared on particular food items, and pair-wise comparisons of these parameters, were studied with the jackknife method (Maia et al. 2000), without adjustment for multiple testing (Webster 2007; Kozak 2009).

## Results

Food source and phytoseiid species significantly affected developmental times of *E. finlandicus* and *T.pyri* (Table 1). The egg stage duration of the mites significantly differed among the food items. On the other hand, on particular prey, embryonic development of *E. finlandicus* and *T.pyri* did not differ (2.7 and 2.4 days on *Te. urticae*; 2.1 and 2.2 days on *S. schizopus*; 2.0 and 2.2 days on *S. garmani*). Only when females of both predators were taken from stock colonies provided with rape pollen, eggs stage duration of *T.pyri* was longer than for *E. finlandicus* (Table 1).

**Table 1:** Mean (±SE) duration (days) of developmental stages and mortality (%) of *Typhlodromus pyri* and *Euseius finlandicus* on a diet of *Tetranychus urticae*, *Schizotetranychus schizopus*, *S. garmani* or rape pollen

Predator species	*Diet*	Egg	Larva	Protonymph	Deutonymph	Egg to adult	Immature mortality (%)
*E. finlandicus*	*Te. urticae*	2.7 ± 0.13 cd	0.9 ± 0.06 d	3.1 ± 0.11 d	3.2 ± 0.16 d	9.9 ± 0.25 c	11.1
	*S. schizopus*	2.1 ± 0.14 ab	2.1 ± 0.07 a	1.7 ± 0.10 ab	2.2 ± 0.08 ab	8.1 ± 0.18 a	7.4
	*S. garmani*	2.0 ± 0.15 a	1.8 ± 0.081 b	1.6 ± 0.09 a	2.2 ± 0.08 ab	7.6 ± 0.14 a	6.7
	Rape pollen	2.6 ± 0.11 ce	1.5 ± 0.10 c	1.8 ± 0.10 ab	2.1 ± 0.10 a	7.9 ± 0.13 a	3.7
*T. pyri*	*Te. urticae*	2.4 ± 0.12 bc	0.9 ± 0.13 d	2.9 ± 0.08 cd	2.5 ± 0.10 b	8.7 ± 0.22 b	7.1
	*S. schizopus*	2.2 ± 0.11 ab	0.7 ± 0.11 d	2.6 ± 0.11 c	2.45 ± 0.16 b	7.9 ± 0.25 a	10.7
	*S. garmani*	2.2 ± 0.12 abe	0.8 ± 0.10 d	2.8 ± 0.09 c	2.9 ± 0.10 c	8.7 ± 0.16 b	6.9
	Rape pollen	3.1 ± 0.15 d	0.9 ± 0.05 d	4.5 ± 0.1 e	4.4 ± 0.17 e	12.9 ± 0.23 d	30.6
Two-way ANOVA (*P* values)	Predator species	0.005	<0.001	<0.001	<0.001	<0.001	
	Diet	<0.001	0.024	<0.001	<0.001	<0.001	
	Predator species * diet	0.54	<0.001	<0.001	<0.001	<0.001	

Means within a column followed by different letters are significantly different (two-way ANOVA followed by Tukey’s multiple comparisons: *P* < 0.05)

The mean duration of larval stage was the highest for *E. finlandicus* feeding on *S. schizopus* (mean ± SE = 2.1 ± 0.07 days). The lowest mean duration of *E. finlandicus* larval stage was obtained on *Te. urticae* (0.9 ± 0.06 days). Larval duration of *T.pyri* reared on particular food items did not differ. The larval developmental period of *T.pyri* was on each tested diet similar to that observed for *E. finlandicus* feeding on *S. schizopus* (ca. 0.8 day). For *T.pyri* the non-feeding larval stage was the shortest developmental phase.

Protonymphs and deutonymphs of *E. finlandicus* fed on *S. schizopus*, *S. garmani* or rape pollen required a significantly shorter period (<2 days) to reach the next stages than *E. finlandicus* fed on *Te. urticae* or *T.pyri* provided with all surveyed items. The highest nymphal duration (over 4 days) was observed for *T.pyri* reared on rape pollen. Overall duration of juvenile developmental time was comparable for *T.pyri* fed *S. schizopus* and *E. finlandicus* reared on *S. schizopus, S. garmani* or rape pollen (ca. 8 days). Duration of development from egg to adult was significantly longest (12.9 ± 0.23 days) when rape pollen was offered to *T.pyri*. Immature mortality was the lowest for *E. finlandicus* fed on rape pollen (mean 3.7 %) and the highest for *T.pyri* on the same food (30.6 %) (Table 1).

Two-way ANOVA showed significant interactions between diet and predator species for pre-oviposition, oviposition and post-oviposition periods (Table 2). Duration of pre-oviposition was shortest when *E. finlandicus* fed on *S. garmani* (2.0 ± 0.51 days) and the longest for *T.pyri* fed on rape pollen (4.4 ± 0.19 days). For the other tested predator-food combinations no differences in pre-oviposition period were observed (ca. 3 days). On average, females of *T.pyri* reared on *S. garmani* oviposited for 28.5 ± 1.49 days. Oviposition time was 3.5× shorter when the same predator was provided with rape pollen (8.1 ± 0.35 days). *Euseius finlandicus* females oviposited for 19 ± 1.54 to 23.1 ± 0.27 days. Post-oviposition of *E. finlandicus* did not differ significantly on particular food items and took around 2 days. *Typhlodromus pyri* post-oviposition time on each tested food was much longer than for *E. finlandicus* (Table 2).

**Table 2:** Mean (±SE) longevity, reproduction and sex ratio of progeny of *Typhlodromus pyri* and *Euseius finlandicus* females on a diet of *Tetranychus urticae*, *Schizotetranychus schizopus*, *S. garmani* or rape pollen

Predator species	Diet	Pre-oviposition (days)	Oviposition (days)	Post-oviposition (days)	Female longevity (days)	Total eggs per female	Eggs per female per day	Sex ratio
*E. finlandicus*	*Te. urticae*	3.1 ± 0.22 c	19.0 ± 1.54 b	2.1 ± 0.27 a	23.7 ± 1.41 a	12.2 ± 0.92 b	0.64 ± 0.05 a	0.75
	*S. schizopus*	3.1 ± 0.16 c	23.1 ± 0.27 b	2.0 ± 0.23 a	28.3 ± 0.80 b	16.5 ± 0.19 c	0.72 ± 0.02 ab	0.65
	*S. garmani*	2.0 ± 0.51 a	22.8 ± 1.27 b	2.6 ± 0.33 a	27.4 ± 1.22 b	21.1 ± 1.08 d	0.90 ± 0.07 cd	0.79
	Rape pollen	3.0 ± 0.21 bc	22.4 ± 0.87 b	2.5 ± 0.29 a	27.9 ± 0.65 b	22.3 ± 0.8 d	1.00 ± 0.05 d	0.73
*T. pyri*	*Te. urticae*	2.9 ± 0.18 bc	23.6 ± 1.21 b	9.1 ± 1.06 b	35.6 ± 1.80 c	19.9 ± 0.93 d	0.80 ± 0.09 bc	0.62
	*S. schizopus*	2.5 +0.23 ab	20.9 ± 0.91 b	15.5 ± 1.17 c	38.8 ± 0.85 c	29.1 ± 0.95 e	1.28 ± 0.11 e	0.63
	*S. garmani*	2.7 ± 0.22 bc	28.5 ± 1.49 c	9.3 ± 1.47 b	40.6 ± 0.95 c	27.5 ± 0.85 e	0.88 ± 0.07 cd	0.77
	Rape pollen	4.4 ± 0.19 d	8.1 ± 0.35 a	9.2 ± 1.17 b	21.7 ± 1.16 a	5.9 ± 0.36 a	0.74 ± 0.04 abc	0.70
Two-way ANOVA (*P* values)	Predator species	0.001	0.001	<0.001	<0.001	<0.001	<0.001	
	Diet	0.023	0.086	<0.001	<0.001	<0.001	<0.001	
	Predator species * diet	<0.001	<0.001	<0.001	<0.001	<0.001	<0.001	

Means within a column followed by different letters are significantly different (generalized linear models, with Poisson distribution of the residuals followed by pair-wise comparisons for combinations of the factors: *P* < 0.05)

When spider mites were offered as food, females of *T.pyri* lived significantly longer than females of *E. finlandicus* (on average 11.9 days longer on *Te. urticae*, 10.5 days longer on *S. schizopus*, and 13.2 days longer on *S. garmani*) (Table 2). Provided with rape pollen, *E. finlandicus* females lived about 6 days longer (27.9 ± 0.65 days) than females of *T.pyri* (21.7 ± 1.16 days).

Fecundity of *E. finlandicus* females (ranging from 12.2 ± 0.92 to 21.1 ± 1.08 eggs per female) was significantly lower than that of *T.pyri* (which ranged from 19.9 ± 0.93 to 29.1 ± 0.95 eggs per female) when predators were fed on the respective spider mite species. On rape pollen, total number of eggs laid by *E. finlandicus* females was about 4× higher than that laid by *T.pyri* (5.9 ± 0.36 eggs per female). Oviposition rate (mean eggs/female/day) was highest (1.3 ± 0.11) for *T.pyri* feeding on *S. schizopus* (Table 2). For *E. finlandicus* oviposition rate was significantly higher on rape pollen than on spider mites (Table 2). Offspring sex ratio did not differ significantly among surveyed predator species reared on the studied diets (Table 2).

Fed on particular spider mite species, *T.pyri* showed higher net reproductive rate (*R*_0_) than *E. finlandicus* (Table 3). On the other hand, when predators were fed on rape pollen, *R*_*0*_ value for *E. finlandicus* (15.7 ± 0.56) was over 5× greater than that for *T.pyri* (2.9 ± 0.18). Mean generation time (*T*) was shortest for *T.pyri* reared on *S. garmani* (20.8 ± 0.45) and *E. finlandicus* fed on *S. schizopus* (20 ± 0.2). The longest mean generation time was observed for *T.pyri* provided with *S. garmani* (25.5 ± 0.71). The intrinsic rate of population increase (*r*_*m*_) and finite rate of population increase (*λ*) were highest when *E. finlandicus* was fed on *S. garmani* (*r*_*m*_ = 0.132, *λ* = 1.14) or *T.pyri* was fed on *S. schizopus* (*r*_*m*_ = 0.136, *λ* = 1.15). The lowest *r*_*m*_ and *λ* were observed for *T.pyri* fed on rape pollen (*r*_*m*_ = 0.05, *λ* = 1.05), whereas the parameters established for *E. finlandicus* fed on pollen were significantly higher than on *Te. urticae* or *S. schizopus* (Table 3).

**Table 3:** Mean (±SE) life table parameters of *Typhlodromus pyri* and *Euseius finlandicus* females on a diet of *Tetranychus urticae*, *Schizotetranychus schizopus*, *S. garmani* and rape pollen

Predator species	Diet	*R*_0_	T	r_m_	*λ*
*E. finlandicus*	*Te. urticae*	8.1 ± 0.61 b	23.1 ± 0.74 c	0.090 ± 0.0022 b	1.09 ± 0.0024 b
	*S. schizopus*	10.0 ± 0.37 c	22.0 ± 0.45 bc	0.105 ± 0.0013 c	1.11 ± 0.0015 c
	*S. garmani*	15.5 ± 0.80 e	20.8 ± 0.45 ab	0.132 ± 0.0013 f	1.14 ± 0.0015 f
	Rape pollen	15.7 ± 0.56 e	22.2 ± 0.40 c	0.124 ± 0.0016 e	1.13 ± 0.0018 e
*T. pyri*	*Te. urticae*	11.5 ± 0.55 d	22.2 ± 0.11 c	0.110 ± 0.0017 d	1.12 ± 0.0019 d
	*S. schizopus*	15.3 ± 1.20 e	20.0 ± 0.20 a	0.136 ± 0.0037 f	1.15 ± 0.0043 f
	*S. garmani*	19.7 ± 0.61 f	25.5 ± 0.71 d	0.117 ± 0.0030 d	1.12 ± 0.0034 d
	Rape pollen	2.9 ± 0.18 a	21.1 ± 0.25 b	0.050 ± 0.0027 a	1.05 ± 0.0028 a

Means within a column followed by different letters are significantly different (Jackknife method: *P* < 0.05)

## Discussion

Both *T.pyri* and *E. finlandicus* developed from egg to adulthood feeding on preys such as *Te. urticae*, *S. schizopus* and *S. garmani* originating from willows. However, the diet affected both biological and demographic parameters of the predators. Only the duration of larval stage of *T.pyri* was similar on the different diets. It seems reasonable because larvae of *T.pyri* usually molt to protonymphs without feeding (Schausberger 1999). Development of the other *T.pyri* and *E. finlandicus* immature stages, interestingly including eggs, was affected by the diet. Differences in egg-stage duration of females provided with different foods could indicate the influence of the maternal diet on eggs development. Such a phenomenon was also reported by Zemek (1993) and Hoffmann et al. (2011).

While feeding on *S. schizopus*—the most common spider mite species occurring on willows (Puchalska et al. 2014)—both tested predators reached adulthood in an equally short time. When *S. garmani* was provided as a food, *E. finlandicus* developed significantly faster than *T.pyri*. The opposite situation was observed during predators’ feeding on juvenile stages of *Te. urticae*. *Tetranychus urticae* was offered as food for *T.pyri* and *E. finlandicus* also by other authors (Kropczyńska 1970; Schausberger 1992; Zhang and Croft 1994; Zemek 1993). Zhang and Croft (1994) showed that overall development time (egg-adult) of *T.pyri* provided with 20 *Te. urticae* eggs per day took 8.73 days; thus it was comparable with that observed in our experiment when the phytoseiid fed on larvae and protonymphs of *Te. urticae* (8.7 days). In the study by Zemek (1993) *T. pyri* fed on diapausing females of *Te. urticae* and reached adulthood after over 19 days. So long developmental time could be explained by a limited opportunity of the sub-adult stages of *T. pyri* to grasp and feed on larger females of the two spotted spider mites (Zemek 1993) than on larvae and protonymps of the pest (i.e., offered in our investigations). Developmental times of *E. finlandicus* provided in our tests with juvenile stages of *Te. urticae* were close to those reported by Kropczyńska (1970) or Schausberger (1992). In turn, Abdallah et al. (2001) stated that overall immature stage duration of *E. finlandicus* feeding on larvae and protonymphs of *Te. urticae* was shorter than that in our study. The differences may be due to different host plants used for rearing spider mites (common bean and willows). Indeed, Koller et al. (2007) and Ferrero et al. (2014) have revealed an indirect, prey-mediated host-plant effect on phytoseiid performance.

Other spider mite species, i.e., *E. carpini* was tested as food for *T. pyri* by Duso and Camporese (1991). During feeding on this prey, predator development (6.3 days) was shorter than on each of tetranychid species tested by us. Still, the direct comparison of *T. pyri* life parameters obtained by Duso and Camporese (1991) and by us is difficult because of different rearing conditions during the experiments. It was previously proved that the temperature may influence developmental and reproductive parameters of Phytoseiidae (Hayes and McArdle 1987; Broufas et al. 2007; Lee and Gillespie 2011; Ganjisaffar et al. 2011; Gadino and Walton 2012).

Several studies indicated a significant role of *T*. *pyri* in controlling *P. ulmi* in vineyards or orchards (Boller et al. 1988; Maixner 1990; Duso 1992a; Camporese and Duso 1996). The importance of *E. finlandicus* in the control of European red mite was also previously described by Van de Vrie (1975), Sechser et al. (1984), Schausberger (1991) and Duso (1992b). Therefore, it is worth to compare life parameters of the predators fed on *P. ulmi* and on spider mites from willows. *Euseius finlandicus* overall development time on *S. garmani* was equal to that reported by Kropczynska (1970) for the predator fed on *P. ulmi*. Moreover, the number of eggs laid by *E. finlandicus* females fed on *S. garmani* was greater than on any other spider mite or eriophyid mite species previously tested (Chant 1959; Kropczyńska 1970; Amano and Chant 1986; Kropczyńska et al. 1988; Schausberger 1992; Abdallah et al. 2001). Nevertheless, the longevity and fecundity of *T. pyri* females were higher on each tested spider mite species than in the case of *E. finlandicus* fed on the same food. The fecundity of *T. pyri* females fed on *S. schizopus* and *S. garmani* was similar to that reported by Sengonca et al. (2003) for *T.pyri* fed on nymphs of *P. ulmi* (31 eggs/overall life span of female). Similar reproductive parameters obtained by the predator on *P. ulmi* and on spider mites from willows indicate a potential role of *T*. *pyri* as biocontrol agent of *S. schizopus* and *S. garmani.* Moreover, higher (or close to) values of r_m_ obtained for *T.pyri* fed on *S. schizopus* (our data) than on *P*. *ulmi* (Overmeer 1981; Lorenzon et al. 2012) encourage future studies on the predator’s effectiveness in this mite’s management.

Both surveyed predatory mites were able to develop also on alternative food, i.e., pollen of *B. napus*. The alternative food may be important for predatory mites in two ways. First, it may help the predator to maintain itself in a locality where spider mites are scarce. Second, it may be of value for rearing predators in laboratory conditions, even if it is not necessarily available to the predator under natural conditions (Overmeer 1985). Pollen can be one of the most valuable alternative food for many phytoseiid species (Addison et al. 2000; Kasap 2005; Bermudez et al. 2010). Its high protein and oil contents make it nutritionally suitable for many arthropods that ordinarily consume prey-food (Lundgren 2009). In some circumstances the presence of pollen can reduce predation rate or search efficiency of phytoseiid mites (Wei and Walde 1997), but very often it is considered as an important factor in Phytoseiidae survival (Overmeer 1985). Our results suggest that rape pollen is suitable as an alternative food especially for *E. finlandicus.* Whereas developmental and demographic parameters of *T. pyri* obtained on rape pollen were much lower than on each spider mite species tested, longevity and fecundity of *E. finlandicus* females observed when fed on pollen was comparable with those obtained on *S. garmani*. Also demographic parameters of *E. finlandicus* indicate that, just after *S. garmani*, rape pollen had the highest nutritional value of surveyed food items. Commercially available rape pollen may be provided on plants as a complementary food. Positive effect of such conservation of indigenous phytoseiid fauna was noticed by Maoz et al. (2011). The pollen can be also used in commercial or small scale mass rearing production systems of *E. finlandicus.*

According to Slone and Croft (2001), *T. pyri* has high mobility and search capacity, thank to which it is able to find complementary food when its prey is scarce. Nevertheless, Kropczyńska-Linkiewicz (1973) revealed that although *T. pyri* was able to develop feeding on pollen, it preferred prey-food. Duso and Camporese (1991) also demonstrated the better response of *T. pyri* to spider mites than to pollen which stays with accordance to our results.

Reassuming, the ability of *T. pyri* and *E. finlandicus* to develop and obtain high values of reproductive and demographic parameters while feeding on *S. schizopus* and *S. garmani* (respectively) suggests their potential role in spider mite control on ornamental willows. Moreover, the capability of *T*. *pyri* of building up resistance to pesticides, even to those compounds that cause high mortality rates such as pyrethroids or organophosphorous insecticides (Overmeer and van Zon 1983; Vidal and Kreiter 1995; Bonafos et al. 2007), gives the opportunity to use the predatory mite in integrated pest management (IPM) conduced in nurseries. Although most authors consider *E. finlandicus* to be quite sensitive to pesticides (Tuovinen and Rokx 1991; Zacharda 2001), it was reported as dominant in the Phytoseiidae complex in organic (Szabó et al. 2014), ecological and IPM orchards (Praslicka et al. 2009). Moreover, the advantage of *E. finlandicus* is the ability to develop on alternative food like pollen. It can be very important especially during spring when other food sources are unavailable. Additionally McMurtry (1992) reported that generalist phytoseiids (like *T. pyri* and *E. finlandicus*) are capable of building up more stable populations that are less dependent on dispersal to new sites in search of prey than specialists. Therefore, our data suggest that both surveyed predatory species, namely *T. pyri* and *E. finlandicus*, have features making them promising biocontrol agents in spider mite control on willows.
